# miR-142-5p promotes cervical cancer progression by targeting LMX1A through Wnt/β-catenin pathway

**DOI:** 10.1515/med-2021-0218

**Published:** 2021-01-28

**Authors:** Lijuan Ke, Yanping Chen, Yiying Li, Zheng Chen, Yihui He, Jiahua Liu, Yingfeng Zhuang

**Affiliations:** Department of Gynecology, Shengli Clinical Medical College of Fujian Medical University and Fujian Provincial Hospital, No. 134 Dong Street, Fuzhou, Fujian 350001, People’s Republic of China; Department of Gynecology and Obstetrics, Shengli Clinical Medical College of Fujian Medical University and Fujian Provincial Hospital South Branch, No. 516 Jinrong South Street, Fuzhou, Fujian 350028, People’s Republic of China; Department of Pathology, Shengli Clinical Medical College of Fujian Medical University and Fujian Provincial Hospital, No. 134 Dong Street, Fuzhou, Fujian 350001, People’s Republic of China; Department of Critical Care Medicine, Shengli Clinical Medical College of Fujian Medical University and Fujian Provincial Hospital South Branch, No. 516 Jinrong South Street, Fuzhou, Fujian 350028, People’s Republic of China

**Keywords:** miR-142-5p, cervical cancer, LMX1A, HeLa cells, Wnt/β-catenin pathway

## Abstract

**Background:**

Previous work has shown that miR-142-5p in cervical cancer tissues increased significantly compared with adjacent normal tissues. However, the function and the mechanism of miR-142-5p in cervical cancer have not been reported.

**Methods:**

Quantitative reverse transcription-polymerase chain reaction (qRT-PCR) was used to determine the gene expression levels. MTT, flow cytometry, and transwell assays were performed to explore the functions of miR-142-5p in HeLa cells. The potential target gene of miR-142-5p was investigated via luciferase reporter assays. The protein expression levels were analyzed by Western blotting.

**Results:**

We found that miR-142-5p expression was elevated but LIM homeobox transcription factor 1 alpha (LMX1A) was decreased in cervical cancer tissues and cells. Overexpression of miR-142-5p or knockdown of LMX1A inhibited cell apoptosis, promoted cell proliferation, migration, invasion abilities, and activated the Wnt/β-catenin pathway. However, knockdown of miR-142-5p or overexpression of LMX1A showed opposite results. LMX1A was identified as a direct target of miR-142-5p by luciferase reporter assays. Finally, rescue experiments demonstrated that LMX1A overexpression attenuated the carcinogenic effect of miR-142-5p mimic on HeLa cells.

**Conclusions:**

These findings suggested that miR-142-5p might be a cervical cancer oncogene and could serve as a potential therapeutic target for the treatment of cervical cancer.

## Abbreviations

HPV, high-risk human papillomavirus
LMX1A, LIM homeobox transcription factor 1 alpha

## 1 Introduction

Cervical cancer is one of the most common gynecologic tumor and the fourth leading cause of death in women with cancer [1]. Approximately 90% of the 2,70,000 cervical cancer deaths in 2015 occurred in low-income and middle-income countries where mortality is 18 times higher than that in developed countries [2]. High-risk human papillomavirus (HPV) infection plays an important role in the pathogenesis of cervical cancer, whereas HPV infection alone is insufficient for development of a cervical cancer [3]. Therefore, elucidating the potential mechanisms in the processes of development and progression of cervical cancer is critical.

MicroRNA (miRNA) is a class of small noncoding single-stranded RNA of 19–24 nucleotides in length, which can downregulate gene expression by binding to the 3′-untranslated regions (3′-UTRs) of target mRNAs [4]. miRNAs are thought to be involved in the development of cancer [5], including cervical cancer [6,7]. Rao et al. [8] used miRNA chip technology to compare differential miRNA expression profiles between cervical cancer tissues and adjacent normal tissues and found that the expression level of miR-142-5p in cervical cancer increased significantly. However, the mechanism of miR-142-5p in cervical cancer has not been reported.

LMX1A (LIM homeobox transcription factor 1 alpha) was indicated as a potential target of miR-142-5p by using Targetscans (http://www.targetscan.org). Liu et al. [9] reported LMX1A can inhibit the metastasis of cervical cancer. Knockdown of LMX1A activated the Wnt/β-catenin-signaling pathway in gastric cancer [10]. Wnt/β-catenin-signaling pathway plays major roles in the occurrence and development of cervical cancer [11]. Therefore, we speculate that miR-142-5p can regulate the development of cervical cancer by targeting LMX1A mediated Wnt/β-catenin-signaling pathway.

## 2 Materials and methods

### 2.1 Clinical samples

Cervical cancer tissues and matched nontumor paraneoplastic tissues were collected from 30 patients who underwent cervical surgical resection. No patients received preoperative radiotherapy, chemotherapy, or other treatment history. All the samples were collected and subsequently snap-frozen in liquid nitrogen at −80°C. This research was approved by the ethics committee of Fujian Provincial Hospital. All patients signed written informed consent forms.

### 2.2 Cell lines, cell culture, and transfection

The human cervical cancer cell line HeLa and normal cervical epithelial cell line Ect1/E6E7 used in this study were purchased from the Shanghai Cell Bank of Chinese Academy of Sciences (Shanghai, China). Cells were cultured in DMEM (Gibco, Grand Island, NY, USA) with 10% FBS in an incubator at 37°C with humidified atmosphere of 5% CO_2_.

For cell transfection, miR-142-5p mimic, miR-142-5p negative control (mimic NC), miR-142-5p inhibitor, anti-miR-142-5p negative control (inhibitor NC), si-LMX1A, and siRNA control (si-NC) were obtained (GenePharma Co, Shanghai, China). The following mimics, inhibitors, and siRNAs were designed and constructed by Zolgene Biotechnology Co., Ltd. (Fuzhou, CHINA): miR-142-5p mimic (sequence: 5′-CAUAAAGUAGAAAGCACUACU-3′); mimic NC (sequence: 5′-UCUACUCUUUCUAGGAGGUUGUGA-3′); miR-142-5p inhibitor (sequence: 5′-AGUAGUGCUUUCUACUUUAUG-3′); inhibitor NC (sequence: 5′-UCUACUCUUUCUAGGAGGUUGUGA-3′); si-NC (sense: 5-UGGAAUAAAUCGAUGUUUGUU-3′ and antisense: 5′-UAAUAAUUCCCAAGAUUUCAC-3′); and si-LMX1A (sense: 5′-GCAAGUAUGACUACGAGAAGC-3′ and antisense: 5′-UUCUCGUAGUCAUACUUGCAG-3′). Cells were plated in 24-well plates before transfection. miR-142-5p mimic and miR-142-5p inhibitor were transfected at a final concentrations of 25 and 50 nm, respectively. The LMX1A gene was synthesized and then cloned into the pcDNA3.1 vector to construct the recombinant plasmid pcDNA-LMX1A. Cell transfections were performed using Lipofectamine 2000 (Invitrogen, Carlsbad, CA, USA) according to the manufacturer’s protocol.

### 2.3 Reverse transcription-polymerase chain reaction (RT-PCR)

Total RNA was extracted from tissues and cells using NucleoZol (Gene Company Ltd, China) following the instructions. Then reverse transcribed using a reverse transcription system, according to the manufacturer’s protocol. qRT-PCR was performed with an ABI 7500 system to detect the expression levels of miR-142-5p and LMX1A. The primer sequences were as follows: miR-142-5p forward: 5′-GCGCGCATAAAGTAGAAAGC-3′, miR-142-5p reverse: 5′-AGTGCAGGGTCCGAGGTATT-3′; U6 forward: 5′-CTCGCTTCGGCAGCACATATACT-3′, U6 reverse: 5′-ACGCTTCACGAATTTGCGTGTC-3′); LMX1A forward, 5′-CGCGCACCCCAACAGA-3′, LMX1A reverse: 5′-GTCGATCGCGCTTTGGAAG-3′; GAPDH forward, 5′-AATGGGCAGCCGTTAGGAAA-3′, GAPDH reverse: 5′-GCGCCCAATACGACCAAATC-3′. U6 or GAPDH was used as an endogenous control. Quantitative PCR thermocycling conditions were: 95℃ for 10 min initially, followed by 35 cycles of 95℃ for 15 s, and 60℃ for 45 s. Relative gene expression was calculated using the 2^–ΔΔCt^ method.

### 2.4 MTT assay

The cells were seeded into 96-well plates at a density of 5 × 10^4^ cells per well and grown at 37°C for 48 h. A total of 20 µL MTT reagent (5 mg/mL) was added to each well. Cells were cultured at 37°C for another 4 h. Then the medium was removed and 150 µL DMSO was added to each well. The absorbance at OD450 nm was determined, and the cell proliferation ability was analyzed.

### 2.5 Evaluation of cell invasion and migration in transwell

For the migration assay, 1 × 10^5^ cells suspended in serum-free medium were seeded into the upper chamber with an 8 µm pore size insert (Corning Incorporated, USA). For the invasion assay, 1 × 10^5^ cells suspended in serum-free medium were seeded into the upper chamber with a Matrigel-coated transwell insert (8 µm pore size). After 24 h of incubation at 37℃, cells that remained on the upper chamber were then removed. The migrating or invading cells on the undersurface of the filters were subsequently fixed by adding methanol and incubated for an additional 30 min. The cells were further stained with crystal violet (0.1%) and incubated for an additional 15 min, and cell counts were obtained for five randomly selected fields of view under a light microscope. All experiments were performed in triplicate.

### 2.6 Evaluation of apoptosis

Apoptosis was detected by flow cytometry analysis of annexin-V staining. After transfection, the cells were harvested and suspended in 300 µL binding buffer. Subsequently, the cells were incubated with 5 µL Annexin-V-FITC and 5 µL PI for 15 min in the dark at room temperature. The apoptosis rate was counted using flow cytometry.

### 2.7 Dual luciferase reporter assay system

The LMX1A 3′-UTR fragment with the putative (LMX1A-WT) or mutated binding site (LMX1A-Mut) of miR-142-5p was inserted into the psiCHECK-2 luciferase reporter vector (Promega, Madison, USA). HeLa cells were grown in a 96-well plate and cotransfected with the vector along with miR-142-5p mimic or negative control. After 36 h of transfection, the relative luciferase activity was measured using a dual-luciferase reporter assay kit (Promega). All experiments were performed independently three times.

### 2.8 Western blot analysis

The collected cells were lysed in PMSF buffer. The protein concentration was determined by using a BCA protein assay kit (Beyotime, China). Protein was separated by SDS-PAGE electrophoresis and transferred to membranes; membranes were subsequently blocked with 5% bovine serum albumin (BSA) at room temperature (RT) for 2 h. Next, the membranes were incubated with anti-LMX1A (ab106629; Abcam, Cambridge, MA, USA), anti-β-catenin (ab6302; Abcam), anti-TCF4 (ab217668; Abcam), and anti-β-actin (Abcam) overnight at 4℃. After washing, the membrane was then probed with specific secondary antibodies (Abcam) at room temperature for 2 h, and ECL visualization was performed. The protein bands were visualized and analyzed using Versa Doc™ imaging system.

### 2.9 Statistical analysis

The data are expressed as mean ± standard deviation (SD). Statistical comparisons between two groups were carried out using Student’s *t* test, and for multiple groups, one-way analysis of variance (ANOVA) was performed using SPSS 20.0 statistical software (SPSS, Inc., Chicago, IL, USA). *P* < 0.05 was considered statistically significant.

## 3 Results

### 3.1 Expression of miR-142-5p and LMX1A in cervical cancer tissues and cells

**Figure 1:**
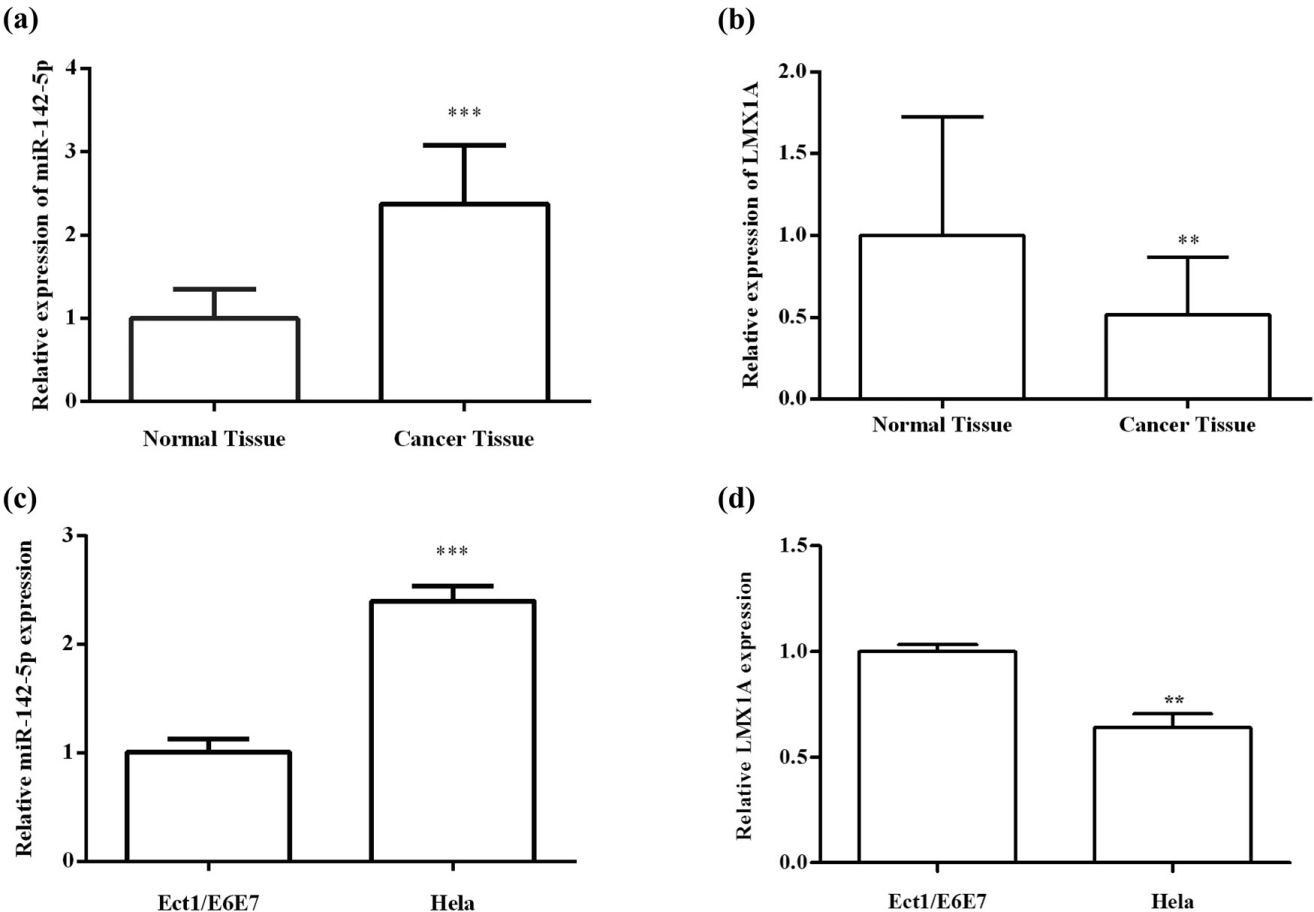
The relative miR-142-5p and LMX1A mRNA expression in cervical cancer tissues and cells were examined by qRT-PCR. Relative miR-142-5p expression levels (a) and LMX1A expression levels (b) in cervical cancer tissues and paracarcinoma tissues; Relative miR-142-5p expression levels (c) and LMX1A expression levels (d) in HeLa and Ect1/E6E7. All experiments were performed in triplicate. ^**^ *P* < 0.01. ^***^ *P* < 0.001.

The relative expression levels of miR-142-5p and LMX1A in cervical cancer tissues and cells were evaluated by qRT-PCR. The results demonstrated that miR-142-5p expression levels were significantly higher (*P* < 0.001) and LMX1A expression levels were significantly lower (*P* < 0.01) in the cervical cancer tissues and cells compared with paracarcinoma tissues and normal cervical epithelial cell line Ect1/E6E7 (Figure 1). These results suggested that miR-142-5p and LMX1A may be related to the development of cervical cancer.

### 3.2 Effects of miR-142-5p on HeLa cell proliferation and apoptosis

**Figure 2:**
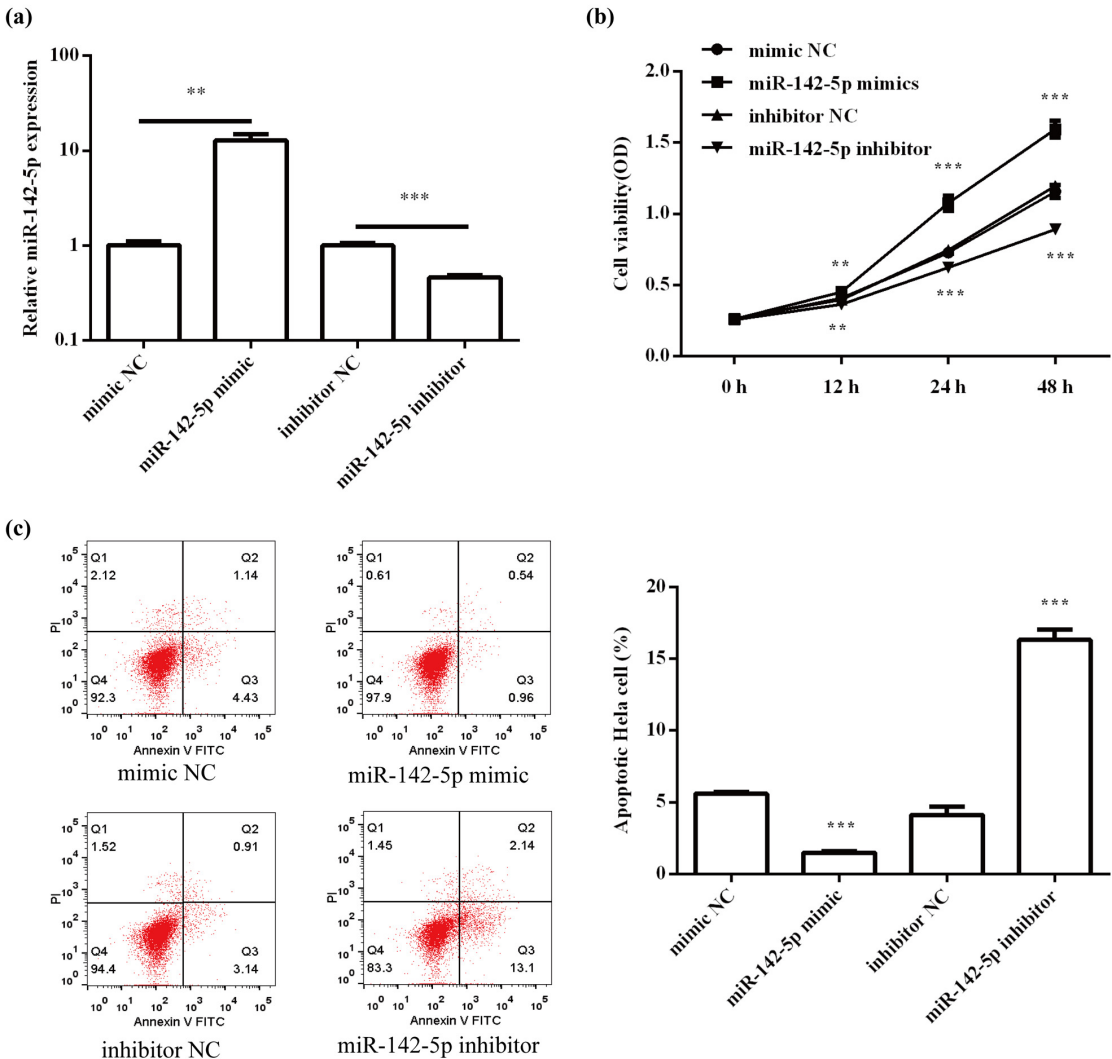
Effects of miR-142-5p on HeLa cell proliferation and apoptosis. (a) The transfection efficiency of miR-142-5p mimic and inhibitor was confirmed by qRT-PCR. (b) The effect of miR-142-5p on cell proliferation was detected by MTT assay. (c) The effect of miR-142-5p on cell apoptosis was assessed by flow cytometry assay. All experiments were performed in triplicate. **P < 0.01. ***P < 0.001.

To determine the effect of miR-142-5p on proliferation and apoptosis, cells were transfected with miR-142-5p mimic or inhibitor to overexpress or knockdown miR-142-5p expression. The transfection effects were confirmed by qRT-PCR. As expected, miR-142-5p expression was elevated in the overexpression group (*P* < 0.01) but was decreased in the knockdown group (*P* < 0.001; Figure 2a). The results of MTT and flow cytometry assay showed that miR-142-5p overexpression remarkably promoted cell proliferation (*P* < 0.001; Figure 2b) and inhibited apoptosis (*P* < 0.001; Figure 2c), while miR-142-5p knockdown significantly inhibited cell proliferation (*P* < 0.001; Figure 2b) and promoted apoptosis (*P* < 0.001; Figure 2c). Overall, miR-142-5p had the ability to affect the proliferation and apoptosis of HeLa cells.

### 3.3 Effects of miR-142-5p on HeLa cell migration and invasion

**Figure 3:**
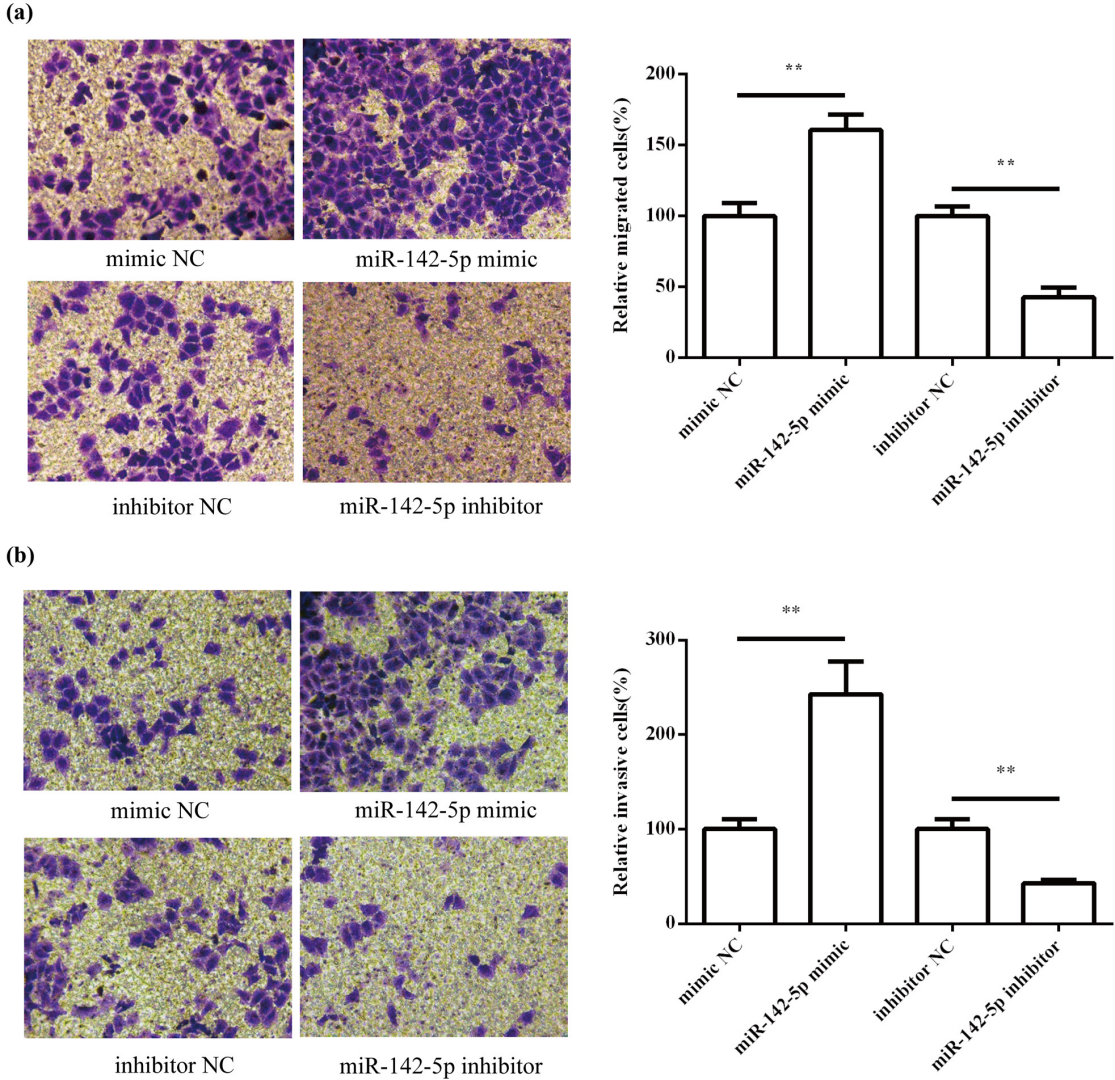
Effects of miR-142-5p on HeLa cell migration and invasion. The effect of miR-142-5p on cell migration (a) and invasion (b) was detected by transwell assay. All experiments were performed in triplicate.^**^ *P* < 0.01.

To investigate whether miR-142-5p regulated the metastasis of HeLa cells, we performed transwell assays to evaluate migration and invasion abilities of HeLa cells. The results showed that the overexpression of miR-142-5p significantly promoted the migration (*P* < 0.01; Figure 3a) and invasion (*P* < 0.01; Figure 3b) of HeLa cells. On the contrary, inhibition of miR-142-5p expression can significantly inhibit HeLa cell migration (*P* < 0.01; Figure 3a)and invasion (*P* < 0.01; Figure 3b).

### 3.4 LMX1A directly targeted miR-142-5p in HeLa cells

**Figure 4:**
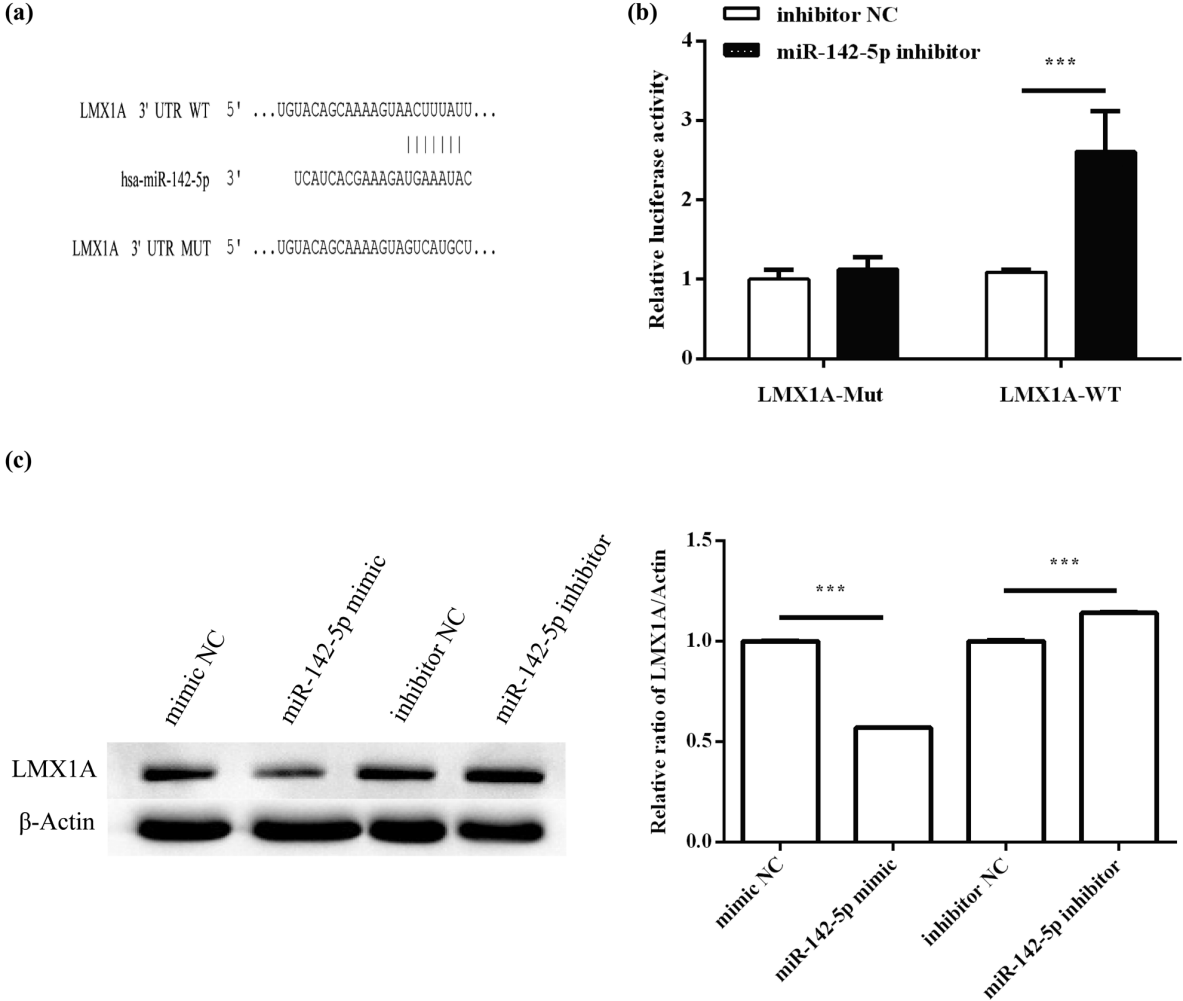
LMX1A is a target of miR-142-5p. a The binding site of LMX1A for miR‑142‑5p was predicted by TargetScan tool. (a) Binding sites for miR-142-5p in the 3′-UTR of LMX1A mRNA. (b) Luciferase reporter assay showing reduced luciferase reporter activity in HeLa cells containing the LMX1A-WT 3′-UTR fragment. (c) Western blotting analysis of expression of LMX1A protein in HeLa cells. All experiments were performed in triplicate. ^***^ *P* < 0.001.

Luciferase reporter gene assay was used for validating that LMX1A was a direct target of miR-142-5p. The wild-type (WT) and mutant (Mut) 3′-UTRs of LMX1A were first constructed (Figure 4a). Subsequently, LMX1A-WT or LMX1A-Mut was cotransfected with the miR-142-5p mimic into HeLa cells. The results showed that when compared with the luciferase activity of the negative control group, the luciferase activity of LMX1A-WT was inhibited markedly (*P* < 0.001), while that of LMX1A-Mut was not suppressed (Figure 4b). Moreover, Western blot analysis revealed that miR-142-5p overexpression dramatically decreased LMX1A protein expression in HeLa cells (*P* < 0.001). However, inhibition of miR-142-5p significantly increased LMX1A protein expression (*P* < 0.001; Figure 4c). Taken together, these findings suggest that LMX1A is a direct target of miR-142-5p in HeLa cells.

### 3.5 Effects of LMX1A on HeLa cell proliferation and apoptosis

**Figure 5:**
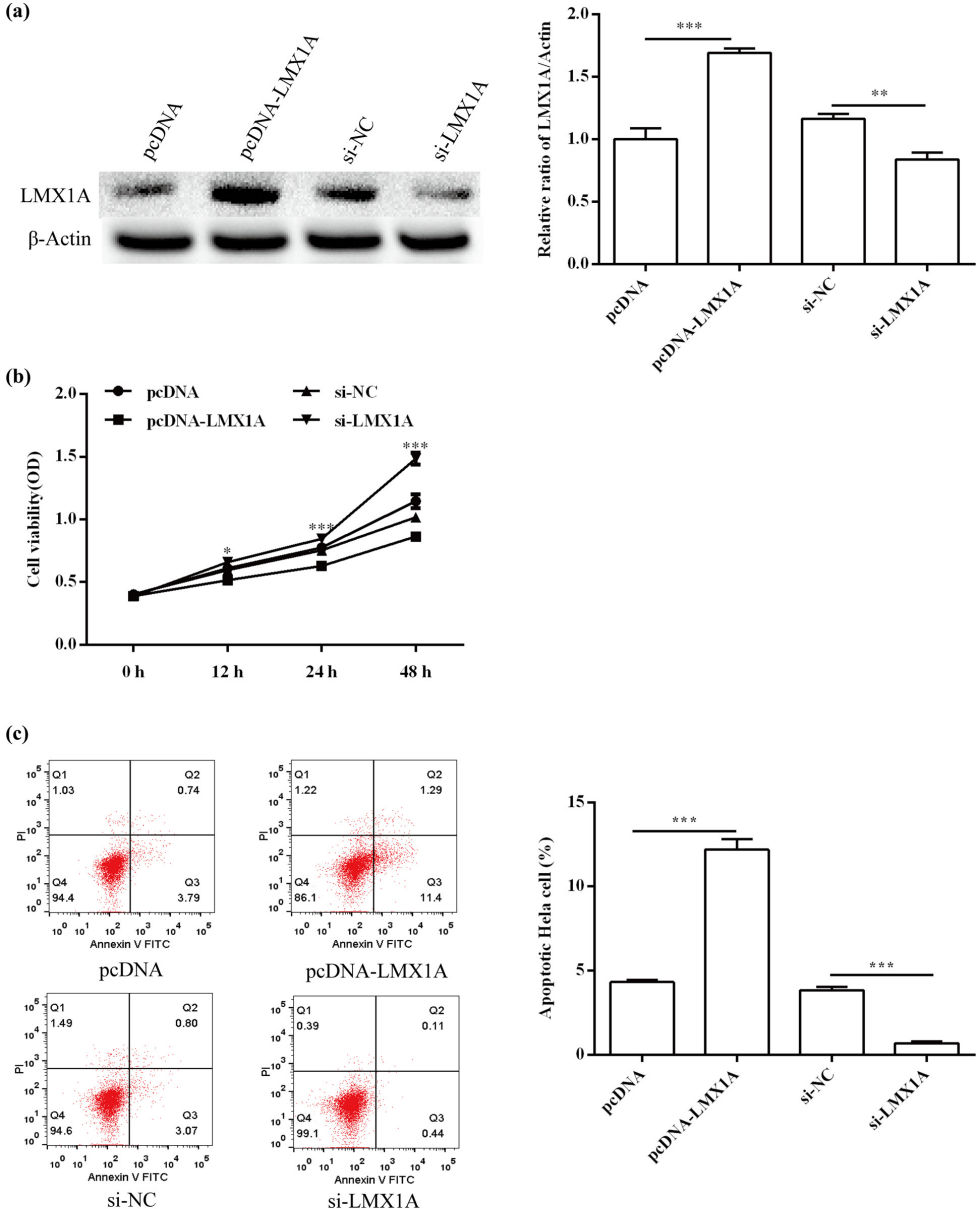
Effects of LMX1A on HeLa cell proliferation and apoptosis. (a) The transfection efficiency of LMX1A mimic and inhibitor was confirmed by qRT-PCR. (b) The effect of LMX1A on cell proliferation was detected by MTT assay. (c) The effect of LMX1A on cell apoptosis was assessed by flow cytometry assay. All experiments were performed in triplicate. ^*^ *P* < 0.05. ^**^ *P* < 0.01. ^***^ *P* < 0.001.

To determine the effect of LMX1A on proliferation and apoptosis, pcDNA-LMX1A and si-LMX1A was used to transfect HeLa cells. The result of qRT-PCR showed that LMX1A expression was elevated in the pcDNA-LMX1A group (*P* < 0.001) but was decreased in the si-LMX1A group (*P* < 0.01; Figure 5a). The results of MTT and flow cytometry assay demonstrated that LMX1A overexpression remarkably promoted cell proliferation (*P* < 0.001; Figure 5b) and inhibited apoptosis (*P* < 0.001; Figure 5c), while miR-142-5p knockdown showed the opposite results (Figure 5b and c). Overall, LMX1A could affect the proliferation and apoptosis of HeLa cells.

### 3.6 Effects of LMX1A on HeLa cell migration and invasion

**Figure 6:**
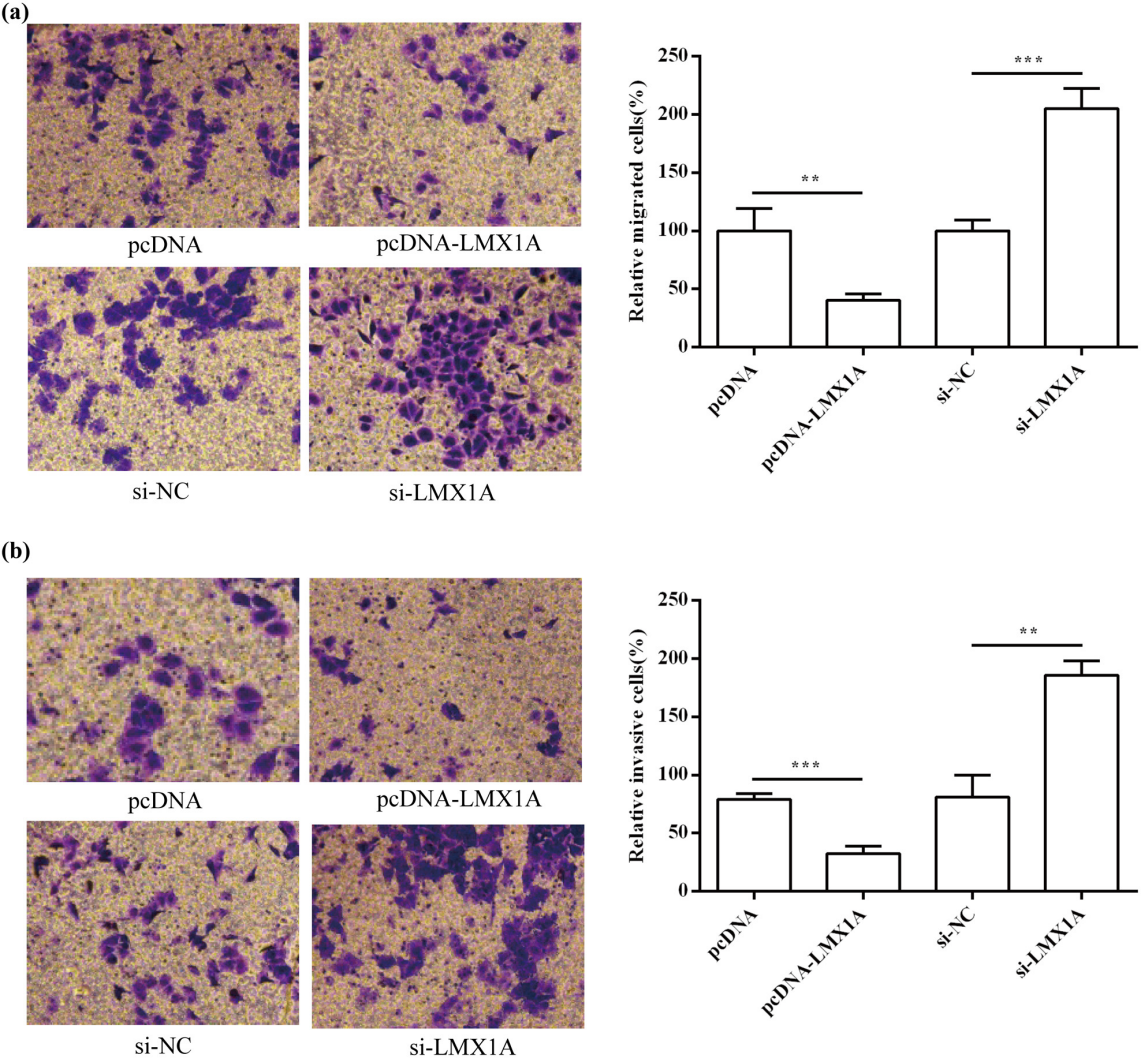
Effects of LMX1A on HeLa cell migration and invasion. The effect of LMX1A on cell migration (a) and invasion (b) was detected by transwell assay. All experiments were performed in triplicate. ^**^ *P* < 0.01. ^***^ *P* < 0.001.

To evaluate whether the expression of LMX1A was able to regulate migration and invasion of cervical cancer cells, we performed transwell assays to determine migration and invasion abilities of HeLa cells. As shown in Figure 6, overexpression of LMX1A inhibited HeLa cell migration (*P* < 0.01) and invasion (*P* < 0.001). Conversely, inhibition of LMX1A expression can significantly promote HeLa cell migration (*P* < 0.001) and invasion (*P* < 0.01).

### 3.7 Effects of miR-142-5p and LMX1A on Wnt/β-catenin-signaling pathway

**Figure 7:**
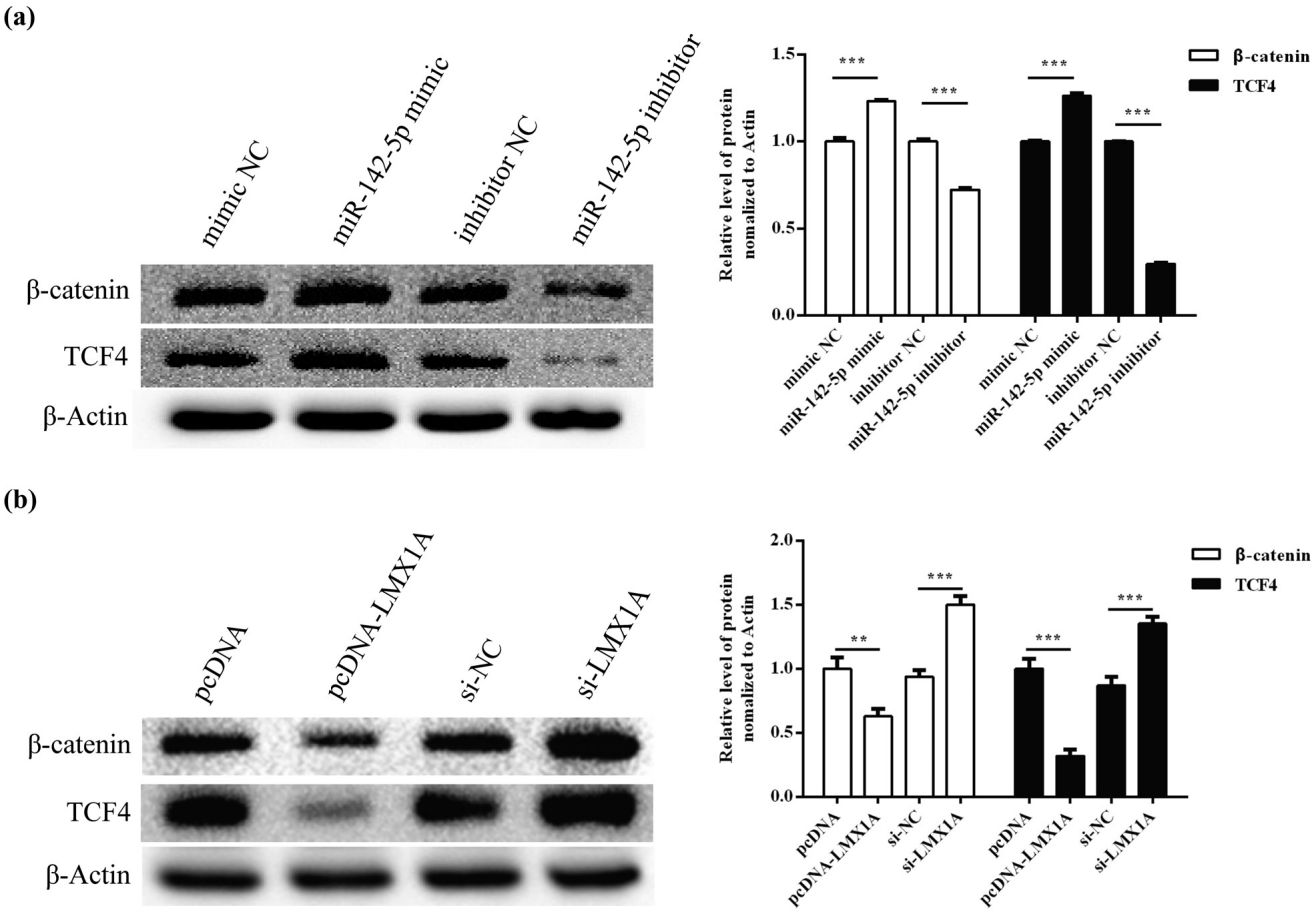
The effect of miR-142-5p and LMX1A on Wnt/β-catenin pathway was assessed by Western blot. (a) Protein expression levels of TCF4 and β-catenin in HeLa cells after transfection with miR-142-5p mimic and miR-142-5p inhibitor. (b) Protein expression levels of TCF4 and β-catenin in HeLa cells after transfection with pcDNA-LMX1A and si-LMX1A. All experiments were performed in triplicate. ^**^ *P* < 0.01. ^***^ *P* < 0.001.

**Figure 8:**
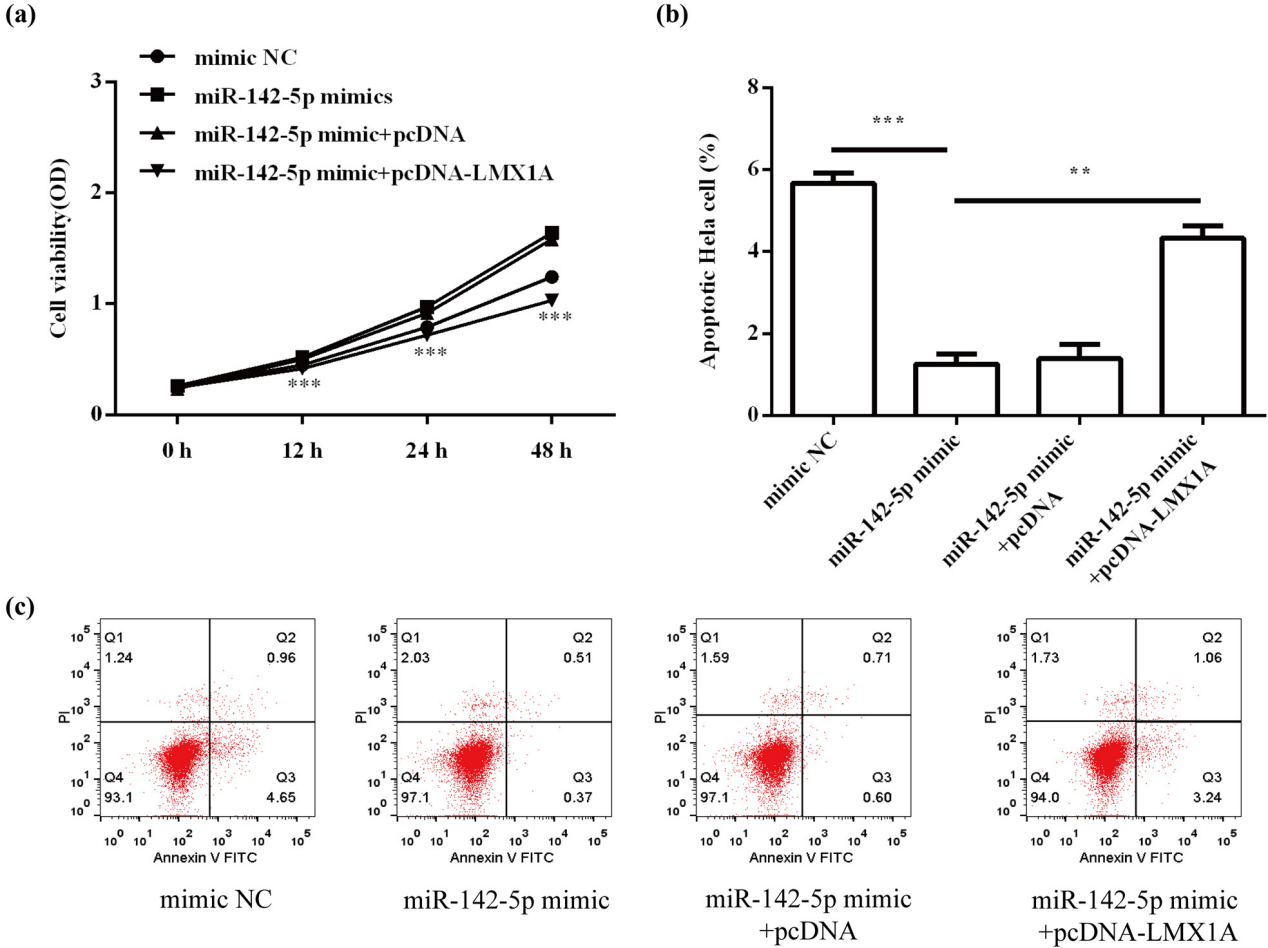
miR-142-5p enhanced HeLa cell proliferation (a) and apoptosis (b) by targeting LMX1A. All experiments were performed in triplicate.^**^ *P* < 0.01. ^***^ *P* < 0.001.

In order to figure out how miR-142-5p and LMX1A participated in the regulation of cell behaviors, the effects of miR-142-5p and LMX1A on the Wnt/β-catenin-signaling pathway were detected. As shown in Figure 8, overexpression of miR-142-5p (Figure 7a) or knockdown of LMX1A (Figure 7b) increased protein levels of TCF4 and β-catenin compared with the control groups (*P* < 0.001). However, knockdown of miR-142-5p (Figure 7a) or overexpression of LMX1A (Figure 7b) significantly decreased protein levels of TCF4 and β-catenin (*P* < 0.001). It was indicated that miR-142-5p reduced LMX1A expression, thereby activating Wnt/β-catenin-signaling pathway in HeLa cells.

### 3.8 miR-142-5p enhanced HeLa cell progression by targeting LMX1A

**Figure 9:**
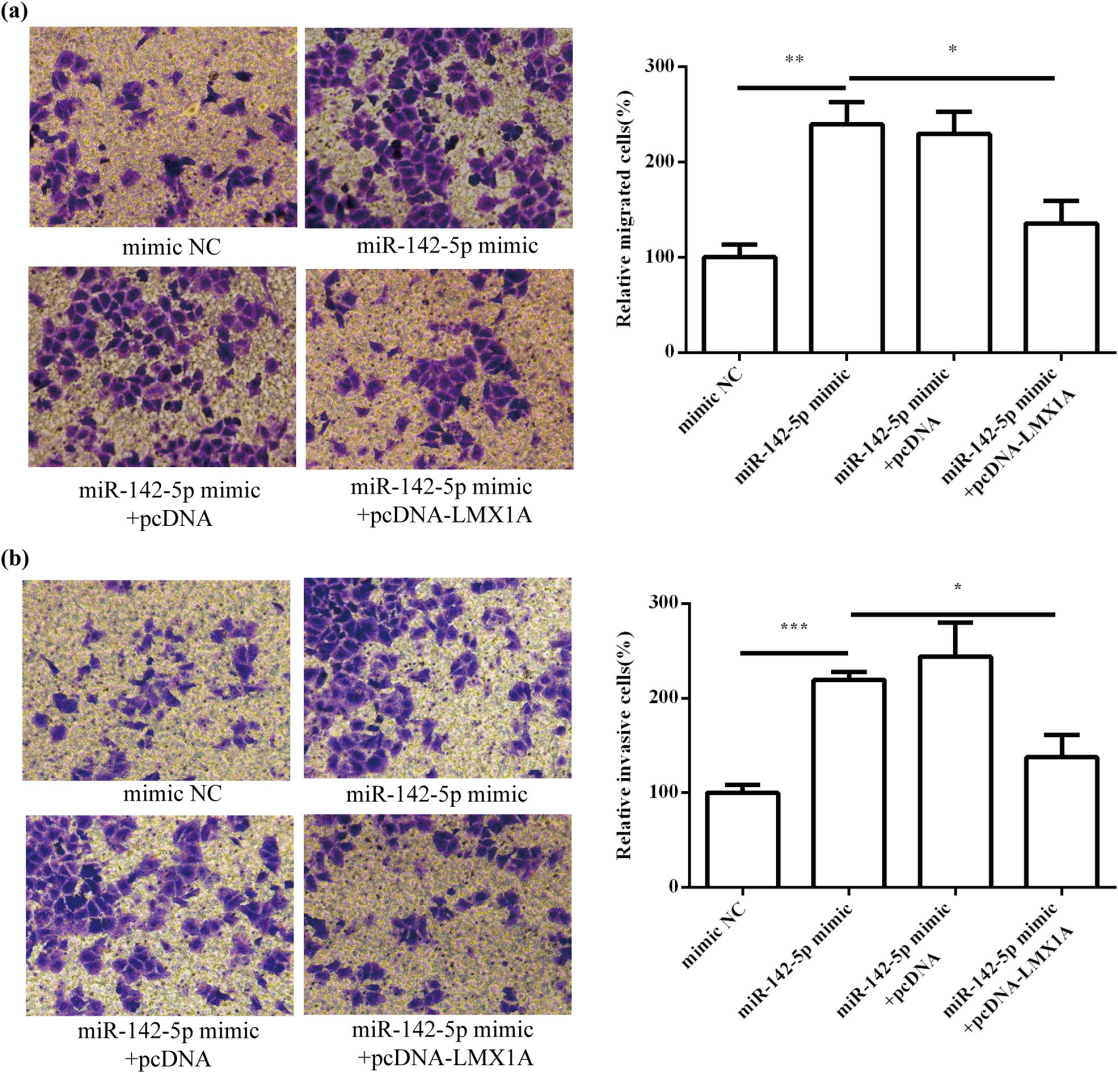
miR-142-5p affected HeLa cell migration (a) and invasion (b) by targeting LMX1A. All experiments were performed in triplicate. ^*^ *P* < 0.05. ^**^ *P* < 0.01. ^***^ *P* < 0.001.

To explore whether LMX1A was involved in the biological roles of miR-142-5p in HeLa cells, miR-142-5p-overexpressing HeLa cells were transfected with pcDNA-LMX1A. The results showed that miR-142-5p mimic promoted cell proliferation (*P* < 0.001; Figure 8a) and suppressed cell apoptosis (*P* < 0.001; Figure 8b), whereas cotransfection of pcDNA-LMX1A alleviated the promotion effect of miR-142-5p on HeLa cell proliferation (*P* < 0.001) and the inhibitory function of miR-142-5p in HeLa cell apoptosis (*P* < 0.01). As shown in Figure 9a and b, overexpression of LMX1 A attenuated the promotion effect of miR-142-5p mimic on migration (*P* < 0.05) and invasion (*P* < 0.05) of HeLa cells. These results suggest that miR-142-5p promotes cervical cell progression by targeting LMX1A.

## 4 Discussion

Emerging studies indicated that miRNAs may have crucial roles in the pathogenesis and progression of various cancers [5]. However, the potential roles of miR-142-5p in the development of cervical cancer are still unclear. Thus, the present study aimed to investigate the role of miR-142-5p in cervical cancer and provide a novel target for the treatment of cervical cancer.

RT-qPCR revealed that miR-142-5p was remarkably increased in human cervical cancer tissues compared with adjacent paired paracarcinoma tissues, which was consistent with the previous study [8]. miR-142-5p was also significantly increased in human cervical cancer tissues and cell lines compared with adjacent paired paracarcinoma tissues and normal cervical epithelial cell line. These data suggested a potential oncogenic role of miR-142-5p in cervical cancer. In this study, miR-142-5p inhibited cell apoptosis, has increased proliferation, invasion, and migration of cervical cell carcinoma cells. These results were similar to the study conducted by Liu et al. [12] and Yao et al. [13]. Therefore, miR-142-5p may regulate tumorigenesis in cervical cancer and act as a promising target for clinical therapy.

Subsequently, the luciferase reporter assay system and Western blot confirmed that miR-142-5p directly targeted LMX1A. LMX1A, an LIM-homeobox gene, is initially known to participate in developmental events [14,15]. LMX1A is mainly considered as a tumor suppressor in various cancers. LMX1A downregulation is associated with ovarian cancer recurrence and poor overall survival [16]. LMX1A knockout promoted gastric cancer cell progression [17]. LMX1A was also identified as a metastasis suppressor in cervical cancer [9]. Similar to these study, the present data demonstrated that LMX1A was decreased in cervical cancer and cell lines, LMX1A induced apoptosis and inhibited proliferation, invasion, and migration of HeLa cells, indicating the potential tumor suppressor role of LMX1A in the development of cervical cancer.

The Wnt/β-catenin-signaling pathway has been reported to be involved in tumorigenesis of a number of types of cancer, including cervical [11,18]. LMX1A negative regulates the Wnt/β-catenin-signaling pathway [10]. This is consistent with our Western blot results that LMX1A inactivated Wnt/β-catenin-signaling pathway. In the present study, Western blotting revealed that miR-142-5p activated the Wnt/β-catenin-signaling pathway. It was indicated that miR-142-5p reduced LMX1A expression, thereby activating Wnt/β-catenin-signaling pathway in HeLa cells. Finally, to explore whether LMX1A was involved in the biological roles of miR-142-5p in HeLa cells, we did the recovery experiment, which confirmed that miR-142-5p could regulate cell proliferation, migration, invasion, and apoptosis by targeting LMX1A.

## 5 Conclusion

Taken together, our study indicated that miR-142-5p may play an onco-miRNA role in the progression of cervical cancer via LMX1A-mediated Wnt/β-catenin-signaling pathway. These findings suggest that miR-142-5p may be a promising target for the development of new treatments for cervical cancer.
